# Pharmacological and therapeutic effects of natural products on liver regeneration-a comprehensive research

**DOI:** 10.1186/s13020-025-01108-y

**Published:** 2025-05-06

**Authors:** Chang Tian, Yuhan Wang, Ran Wang, Linxin Pan, Tao Xu

**Affiliations:** 1 Inflammation and Immune Mediated Diseases Laboratory of Anhui Province, School of Pharmaceutical Sciences, Anhui Medical University, Hefei, 230032 China; 2 Anhui Key Lab of Bioactivity of Natural Products, Institute for Liver Diseases of Anhui Medical University, Anhui Medical University, Hefei, 230032 China; 3 International Cooperation and Exchange Department, Shanghai General Hospital, 85/86 Wujin Road, Hongkou District, Shanghai, 200434 China; 4 College of Life Sciences, Anhui Medical University, Hefei, 230032 China

**Keywords:** Natural products, Liver regeneration, Traditional Chinese medicine

## Abstract

Liver regeneration (LR) refers to the physiological process by which hepatocytes undergo cellular proliferation to restore the structure and function of the liver following significant hepatocyte loss due to injury or partial hepatectomy (PH). While the liver possesses a remarkable regenerative capacity, this process is tightly regulated to ensure appropriate cessation once homeostasis is reestablished. Various strategies, including technological interventions and pharmacological agents, have been explored to enhance LR. Among these, natural products have emerged as promising candidates for promoting LR. For instance, quercetin, a natural compound, has been shown to enhance LR following PH by maintaining redox homeostasis and stimulating hepatocyte proliferation. However, natural products present certain limitations, such as poor solubility and low bioavailability, which may hinder their clinical application. Modifications in the formulation and mode of administration have demonstrated potential in overcoming these challenges and optimizing their pharmacological effects. Recent advancements in research have further highlighted the growing relevance of natural products, including traditional Chinese medicine (TCM), in the context of LR. Despite this progress, a comprehensive and systematic review of their roles, mechanisms, and therapeutic potential remains lacking. This review aims to bridge this gap by summarizing natural products with demonstrated potential to promote LR. Drawing on data from PubMed, Web of Science, and CNKI databases, it elucidates their pharmacological effects and regulatory mechanisms, providing a valuable reference for future research and clinical application in the field of LR.

## Introduction

Liver regeneration (LR) refers to the liver's ability to restore its structure and function following partial hepatectomy (PH) or chemical injury. This process is primarily driven by hepatocyte proliferation, supported by other cell types such as biliary epithelial cells, which can differentiate into hepatocytes and contribute to tissue repair. Promoting LR is clinically significant; for instance, effective recovery from liver transplantation (LT) relies on robust regenerative capacity. According to 2023 statistics, about 32.4% of the world's population is affected by non-alcoholic fatty liver disease (NAFLD), and the number of deaths has doubled in the last three decades (9,3757 in 1990 and 16,8969 in 2019), has become an important reason of LT in Europe (8.4% of liver transplants in 2016) and United States (21% of liver transplants in 2018) [1], and after LT the transplanted liver rapidly starts the regeneration process to adapt to the needs in the recipient's body, so excellent liver regenerative capacity helps the transplanted liver to regain its function more quickly. Liver regenerative capacity can be enhanced by traditional pharmacological interventions or novel mesenchymal stem cell transplantation techniques [2] and the establishment of decellularized liver scaffolds [3], etc. Among these, natural product-derived drugs have garnered significant attention due to their favorable safety profile and abundant availability, offering promising potential in the field of LR research.

Natural products refer to organic compounds with specific chemical structures and biological activities produced by plants, animals, and microorganisms, which can be divided into flavonoids, alkaloids, polysaccharides, saponins, etc., and have the advantages of wide sources and low toxicity. In addition, their excellent pharmacological effects have also been under close scrutiny by scientists, such as anti-inflammatory, antioxidant [4, 5], antimicrobial [6, 7], antiviral [8, 9], and immunomodulatory effects [10, 11], these outstanding pharmacological effects provide the feasibility of natural products in treating cancer [12], viral diseases [13] and cardiovascular diseases [14]. In addition, natural products gerberellin [15] and silymarin [16], which are natural hepato-protective agents, have shown therapeutic effects on liver diseases. Meanwhile, Geraniol (Ger) and dioscin are also able to promote LR by promoting hepatocyte proliferation and reducing liver injury. Notably, in addition to natural product monomers, Chinese herbal compound preparations also showed excellent effects in promoting LR. For example, SiNiSan (SNS), a traditional formula, was able to promote hepatocyte proliferation by resisting oxidative stress and facilitated LR in mice after 70% PH [17]. All these results suggest that natural products have great potential in promoting LR.

Collectively, accumulated evidence indicates that natural products exert a promotive effect on LR. This review provides a comprehensive summary of natural product monomers with the potential to enhance LR, along with their underlying regulatory mechanisms, aiming to support drug discovery efforts in this domain.

## Overview of LR

LR refers to the process that after PH or liver damage, the number of hepatocytes decreases dramatically, and a variety of feedback signals can promote the proliferation of hepatocytes in the G0 phase, and the residual hepatocytes compensate for the loss of and damage to liver tissues and restore the liver's physiological functions through cell proliferation. The organism possesses the ability to precisely detect the size of the regenerating liver and halt LR at the appropriate time.

**Fig. 1:**
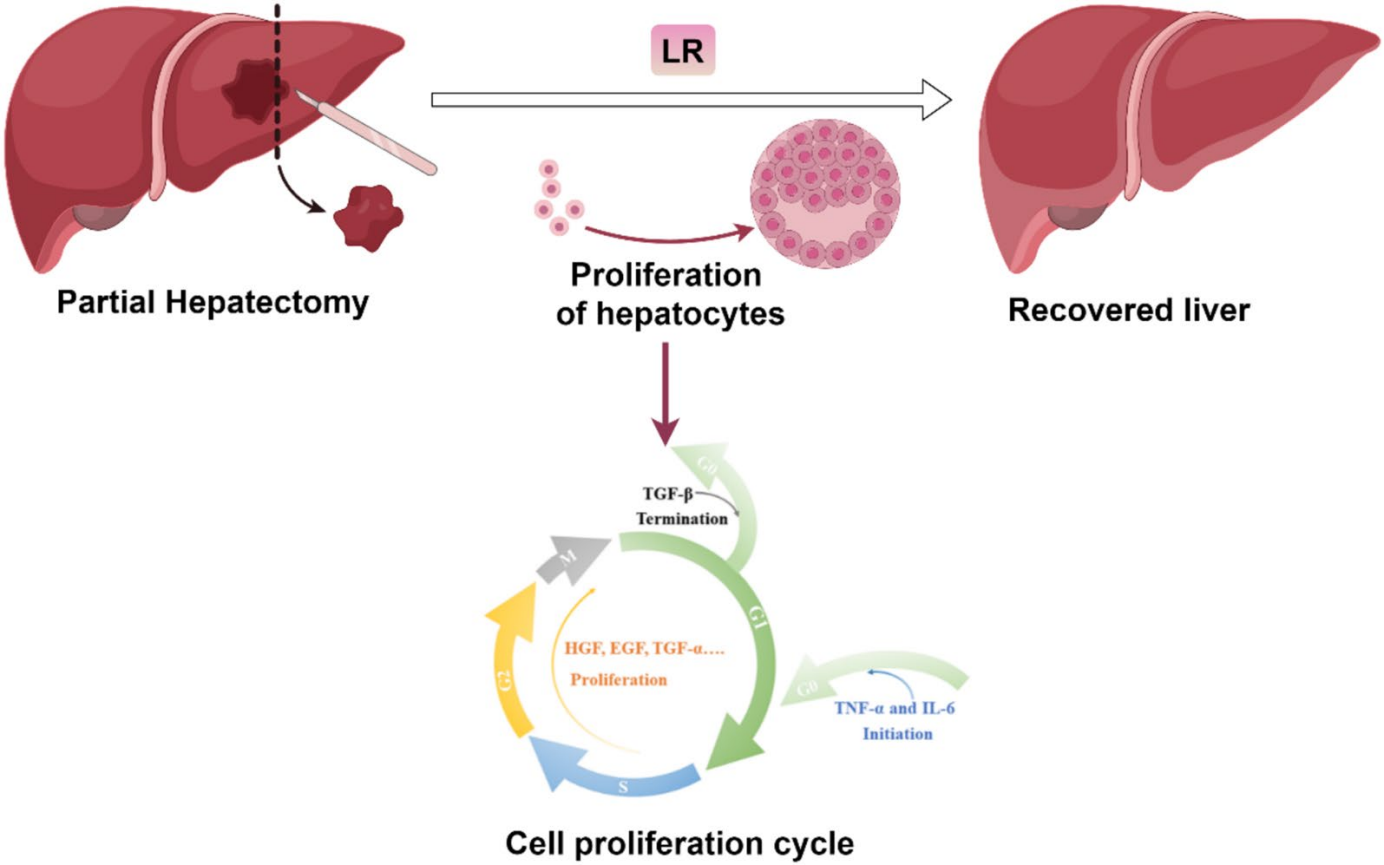
The outline of the LR process. After PH, the remaining hepatocytes undergo three phases, including priming phase, proliferative phase and termination phase, and a number of factors including IL-6, TNF-α, EGF, HGF, TGF-α, BA, NE, oestrogen, VEGF, and TGF-β are involved in this process, which collectively help in the recovery of the liver. *LR* liver regeneration, *PH* partial hepatectomy, *IL-6* interleukin-6, *TNF-α* tumor necrosis factor-α, *EGF* epidermal growth factor, *HGF* hepatocyte growth factor, *TGF-α* transforming growth factor-α, *BA* bile acids, *NE* norepinephrine, *VEGF* vascular endothelial growth factor, *TGF-β* transforming growth factor-β

LR consists of three phases, including priming phase (the first stage), proliferative phase (the second stage), and termination phase (the third stage) (Fig. 1). The first (I) stage is priming phase, in which interleukin-6 (IL-6) and tumor necrosis factor-α (TNF-α) are key factors that stimulate the transition of hepatocytes from G0 phase to the G1 phase of the cell cycle [18]. Subsequently, the second (II) stage is proliferative phase, in which complete mitogens and auxiliary mitogens play an important role. Complete mitogens such as epidermal growth factor (EGF), hepatocyte growth factor (HGF), and transforming growth factor-α (TGF-α), can directly stimulate DNA synthesis and cell proliferation. Moreover, accessory mitogens include bile acids (BA), norepinephrine (NE), estrogen, and vascular endothelial growth factor (VEGF), which can promote hepatocyte proliferation by amplifying or accelerating the effects of full mitogens and may also lead to delayed LR in their absence [19]. Stimulated by these cytokines, hepatocytes complete mitosis. Finally, the third (III) stage is termination phase, transforming growth factor-β (TGF-β) is a negative regulatory factor, which inhibits hepatocyte mitosis and proliferation, stopping LR [20]. After going through these three stages, the liver completes regeneration.

Several factors influencing the process of LR warrant attention. First, hepatic portal artery hemodynamics play a pivotal role. Studies have demonstrated that increased portal blood flow and pressure can stimulate LR by activating HGF, promoting hepatocyte hypertrophy, and inhibiting apoptosis [21]. Second, the accumulation of acetylcholine has been found to enhance LR and protect the liver from surgery-induced injury via the action of M3 muscarinic acetylcholine receptor antagonists [22]. Finally, autophagy, a process of breaking down intracellular components through lysosomes, maintains and regulates homeostasis within eukaryotic cells [23]. Reduced autophagic activity significantly impairs LR after PH, whereas enhancing autophagy through mTOR-independent inducers markedly improves LR in aging livers [24]. Collectively, these findings have guided the identification of several potential therapeutic agents targeting LR, offering promising avenues for the treatment of liver diseases.

Currently, while various drugs have been identified to promote LR, natural products have demonstrated distinct advantages in recent years, highlighting the need for further exploration of their potential in facilitating LR.

## Single-component natural products for LR

Currently, studies have proved that natural product monomers have excellent performance in treating liver diseases, such as resveratrol (RESV) for NAFLD [25] and silymarin for hepatic fibrosis [26]. In addition, the effects of natural product monomers on LR have been well-documented, including those derived from saponins, flavonoids, phenols, and terpenoids (Table 1).

**Table 1:** The role of single components of natural products in promoting LR

Categories	Monomers	Chemical structures	Effects
	Dioscin		ALT, AST↓ PCNA↑ Notch1↑ Jagged1↑ Cyclins↑ CDK4, CDK2↑
Saponin	Panax notoginseng saponins		ALT, AST↓ PCNA↑ PI3K, Akt↑ mTOR ↑
	Baicalin		ALT, AST↓ PCNA↑ cyclin D1↑ IL-1β, IL-18↑
Flavonoid	Dihydromyricetin		ALT, AST↓ SOD↑ PCNA↑ IL-1β, IL-6, TNF-α↓ cytochrome c↓ caspase -8、3、6、9↓
	Quercetin		PCNA↑ SOD, GSH↑ MDA↓
Phenols	Curcumin		PCNA↑ SOD, GSH↑ MDA↓
	Resveratrol		ALT, AST↓ CYP2E1, CYP3A11,CYP1A2↓ PCNA↑ CyclinD1↑ CDK4↑
	Schisandrol B		HGF, EGF↑ IL-6↑ cyclinD1↑ PCNA↑ P53, p21↓ Bcl-2↑
	Rosmarinic acid		ALT, AST ALP↓ PCNA↑
Terpenoids	Ursolic acid		DNA↑ CyclinD1↑ CyclinE↑ C/EBPβ↑
	Oleanolic acid		PCNA↑ CyclinD1↑ CyclinE1↑ FOXO, RBL2, CDKN1B↓
	Geraniol		ALT↓ TNF-α, IL-6↑

### Saponins

Dioscin, a natural steroidal saponin, mainly presenting in andrographis paniculata of the dioscoreaceae family, exhibits various pharmacological effects such as anti-inflammatory, antioxidant [27], and anti-apoptosis [28], and has been shown to treat NAFLD [29], relieve acetaminophen (APAP) -induced liver injury [30], and improve hepatic ischemia–reperfusion (I/R) injury [31]. In addition, dioscin had a positive effect on LR, as evidenced by the ability of dioscin to promote recovery and regeneration of the liver in the 70% PH mice model [32]. Alanine aminotransferase (ALT) and aspartate aminotransferase (AST) are well-recognized biomarkers for assessing liver injury, with elevated serum levels indicating hepatic damage. Recent studies have demonstrated that dioscin effectively reduces serum ALT and AST levels, thereby mitigating liver injury in mice subjected to 70% PH [33]. Furthermore, proliferating cell nuclear antigen (PCNA) is an important cofactor for DNA replication [34], and the cell cycle proteins (Cyclins), as well as cell cycle protein-dependent kinases (CDKs), are at the core of cell cycle regulation [35], all of which are crucial indicators for evaluating the proliferative state of cells. It is well-documented that dioscin significantly enhances the expression of PCNA, cyclin-dependent kinase 4 (CDK4), cyclin-dependent kinase 2 (CDK2), cell cycle protein D1 (Cyclin D1), and cell cycle protein E1 (Cyclin E1) in primary hepatocytes and AML-12 cells in mice, thereby promoting cell proliferation and LR [32]. In summary, dioscin is widely used as a highly potent and low toxicity natural hepatoprotective agent for the treatment of various liver diseases, in which its effects on LR are also outstanding, not only promoting LR through direct stimulation of hepatocyte proliferation but also reducing liver injury and providing favorable conditions for LR. However, dioscin has low solubility, poor stability [36], and poor oral bioavailability [37] limiting its clinical application. Researchers have designed hybrid micelles consisting of D-α-Tocopherol polyethylene glycol 1000 succinate (TPGS) and Soluplus® copolymers encapsulating insoluble dioscin to improve its solubility and stability [36], it provides more possibilities for the clinical use of dioscin for LR.

Panax notoginseng saponin (PNS) is a kind of saponin natural product extracted from the traditional Chinese herb Panax notoginseng, which possesses anti-inflammatory [38] and anti-oxidative stress [39] effects. PNS has shown efficacy in treating NAFLD [40], preventing hepatic fibrosis [41], and alleviating alcoholic liver disease (ALD) [42]. Recent studies have indicated that PNS has the potential to promote LR. In the 70% PH mouse model, PNS was able to reduce the levels of serum ALT and AST, alleviate liver injury, and increase the expression of PCNA, which significantly promotes the proliferation of primary mouse hepatocytes. Moreover, PNS can reduce apoptosis in primary mouse hepatocytes and facilitate the regeneration of damaged livers [43]. In summary, PNS contributes to LR by promoting hepatocyte proliferation and inhibiting hepatocyte apoptosis. However, careful consideration should be given to dosage and individual patient characteristics to minimize the risk of adverse effects.

### Flavonoids

Baicalin, a flavonoid natural product derived from scutellaria baicalensis, exhibits remarkable pharmacological effects such as antioxidant [44], anti-inflammatory [45], and lipid metabolism regulation [46]. As the study has progressed, baicalin has shown hepatoprotective effects in hepatic fibrosis [47], ALD [48], and NAFLD [49]. Meanwhile, recent studies have shown that in the APAP-induced liver injury mice model, baicalin significantly decreased serum ALT and AST levels, and increased the expression of PCNA and Cyclin D1, promoting hepatocyte proliferation [50]. In addition, interleukin-18 (IL-18) is a cytokine that can promote the proliferation of hepatocytes after hepatectomy and is beneficial for LR [51], NOD-like receptor pyrin domain containing 3 (NLRP3) inflammasome, a multimeric protein complex that stimulates the generation of interleukin-1β (IL-1β) and IL-18 and is a target of natural products to alleviate liver injury [52]. Notably, it has been reported that NLRP3 is closely associated with the regeneration of a variety of organs or tissues, including epithelial regeneration of lung tissue [53], bone regeneration [54], skin regeneration [55], nerve regeneration [56]. Additionally, its role in liver tissue regeneration has also been confirmed [57]. Existing literature has thoroughly demonstrated that baicalin can activate NLRP3, leading to increased levels of IL-1β and IL-18 in the liver, thereby promoting hepatocyte proliferation [50]. These findings have confirmed that baicalein can be used to promote LR, and its wide range of sources, and low toxicity [58] give it great potential and advantages in the field of liver disease treatment.

Dihydromyricetin (DMY) is a flavonoid mainly found in Garcinia cambogia, a vine plant, and is a natural antioxidant [59] with anti-inflammatory effects [60]. DMY has been used in treating NAFLD [61] and APAP-induced liver injury [62], and it also promotes LR. In the CCl_4_-induced acute liver injury mice model, DMY reduced inflammation and oxidative stress in the liver by attenuating the inflammatory response, lowering serum levels of ALT and AST, and enhancing superoxide dismutase (SOD) activity [63]. Additionally, apoptosis-related proteins Caspase family and cytochrome c, are essential for the initiation and execution of apoptosis [64, 65], and the literature has amply demonstrated that DMY can reduce hepatocyte apoptosis by inhibiting the expression of cytochrome c and Caspase -8, 3, 6 and 9. At the same time, DMY increased the expression of PCNA, which promotes hepatocyte proliferation [63]. These findings suggest that DMY is characterized by a synergistic effect of anti-inflammatory, antioxidant, hepatocyte proliferation, and apoptosis reduction to promote LR after liver injury.

Quercetin (Que) is a polyphenolic flavonoid found in the stem bark and fruit skin of plants, and its anti-inflammatory [66], antioxidant [67], and anti-apoptotic effects [68] have been demonstrated in studies. Que, as a natural hepatoprotective agent, its efficacy in liver disease is prominent, such as NAFLD [69], hepatic fibrosis [70], and alcohol-induced liver injury [71]. In addition, it was shown that Que also has a prominent effect on LR, in the 70% PH rats model, Que can increase the expression of PCNA, promote hepatocyte proliferation, and significantly reduce hepatocyte apoptosis after PH [72]. In addition, maintaining redox balance in the liver can promote LR [17], Malondialdehyde (MDA) is an important biomarker for assessing lipid peroxidation [73]. Additionally, SOD [74] plays a key role in antioxidant defense, while glutathione (GSH) [75] is an important antioxidant in the body. GSH helps regulate reactive oxygen species (ROS) levels in vivo, thereby reducing cellular damage associated with lipid peroxidation. The study clearly demonstrates that Que can reduce MDA levels in the liver while increasing GSH levels and SOD activity, thereby exerting antioxidant effects and promoting LR [72]. In summary, Que has been demonstrated as a natural hepatoprotective agent and can promote LR after PH.

### Phenols

Curcumin (CUR), a phenolic natural product found in turmeric, with biological activities such as anti-inflammatory [76], antioxidant [77], and antibacterial [78], and has been shown to alleviate various liver diseases, such as hepatic fibrosis [79], NAFLD [80], and drug-induced liver injury [81]. Meanwhile, in the 70% PH rat model, CUR was able to promote LR. CUR exhibited antioxidant activity that can reduce the level of MDA and increase the levels of SOD activity and GSH in liver tissues. Meanwhile, CUR also increased the expression of PCNA to promote hepatocyte proliferation and reduced apoptosis in hepatocytes [82]. Currently, there are limited reports on the mechanisms through which CUR promotes LR, highlighting the need for further investigation into its potential for enhancing LR. However, the phenolic structure of CUR results in very low solubility, poor stability, and low bioavailability, which greatly hampers the development of its clinical applications, to ameliorate these limitations, some improvements can be made to its delivery format to facilitate its clinical application in LR.

RESV, a phenolic compound found mainly in grapes, exhibits anti-inflammatory [83], anti-tumor [84], and other pharmacological effects. Furthermore, RESV has been reported to be effective in alcoholic fatty liver disease (AFLD) [85], NAFLD [25], and hepatic fibrosis [86]. In addition, RESV has shown the potential to promote LR. Firstly, RESV can reduce liver injury, it significantly suppressed cytochrome P450 (CYP450) activity, especially CYP2E1, CYP3A11, and CYP1A2, thus reducing the production of hepatotoxic N-acetyl-p-benzoquinone imine (NAPQI), the serum ALT and AST levels were also reduced, and the use of RESV pretreatment increased GSH levels and reduced oxidative stress, all of which indicated that APAP-induced liver injury was attenuated with RESV. Secondly, RESV increased the levels of PCNA, as well as the cell cycle regulatory proteins CyclinD1 and CDK4, which promoted the proliferation of hepatocytes [87]. In a word, RESV’s high safety profile and wide availability underscore its potential to promote LR in future applications.

Schisandrin B (SolB) is a phenolic natural compound derived from the herb Schisandra chinensis. It exhibits significant pharmacological properties, including anti-aging and anti-inflammatory effects [88]. As a natural hepatoprotective agent, SolB is able to treat acute hepatotoxicity caused by APAP [89], as well as cholestatic liver injury [90]. After liver injury, Kupffer cells secrete IL-6, an inflammatory cytokine that induces hepatocyte dedifferentiation and thus promotes LR [91], in addition, HGF and EGF [92] are factors that can promote LR. Experiments have shown that in the 2/3 PH mice model, SolB increased the levels of IL-6, HGF, and EGF, induced the expression of Cyclin D1 and PCNA, and promoted LR [93]. Moreover, p53 is an important tumor suppressor gene and its activation leads to cell cycle arrest, cellular senescence, and even cell death [94]. P21, a known cell cycle protein-dependent kinase inhibitor, is mainly transcriptionally regulated by p53 [95], and both are capable of adversely affecting LR. It should be mentioned that in the APAP-induced liver injury model, SolB was able to reduce the expression of p53 and p21 [96]. In addition, B-cell lymphoma-2 (Bcl-2) family proteins are able to influence apoptosis by controlling the permeability of the mitochondrial outer membrane, and this family can be categorized into pro-apoptotic proteins and anti-apoptotic proteins (e.g. Bcl-2) [97]. Notably, it is well documented that SolB can increase the expression of Bcl-2, decrease hepatocyte apoptosis, and have positive effects on LR [96]. Therefore, SolB represents a promising therapeutic option for enhancing LR, particularly in the context of liver recovery following partial hepatectomy (PH) or liver transplantation.

Rosmarinic acid (RA), a phenolic acid ingredient primarily found in rosemary, with anti-inflammatory, antioxidant, anti-apoptotic [98], and antimicrobial [99] effects, has been shown to treat APAP-induced liver injury [100] and nonalcoholic steatohepatitis (NASH) [101]. In addition, RA has the potential to promote LR. In the 2/3 PH mouse model, RA treatment reduced serum levels of alanine ALT, AST, and alkaline phosphatase (ALP). Furthermore, RA increased the expression of PCNA, promoted hepatocyte proliferation, and alleviated liver injury [102]. These results suggest that RA is a promising natural product that enhances LR. However, further investigation is needed to fully understand the role of RA in LR, and the safety of its clinical application requires additional research.

### Terpenoids

Ursolic acid (UA), a terpenoid found mainly in bearberry, exhibits strong pharmacological activities such as anti-inflammatory [103], antibacterial, and antioxidant [104], and UA is effective in a number of liver diseases, such as NAFLD [105] and hepatic fibrosis [106]. Meanwhile, UA exhibited a remarkable ability to promote LR. In the 70% PH mouse model, UA significantly enhanced DNA synthesis in hepatocytes. Additionally, the expression of Cyclin D1, cyclin E, and CCAAT/enhancer-binding protein β (C/EBPβ) was elevated. C/EBPβ is a protein known to facilitate LR following activation, suggesting that UA may promote hepatocyte proliferation and support LR after 70% PH [107]. However, UA has shown shortcomings such as low bioavailability and solubility, poor targeting, and rapid metabolism, and has certain limitations in clinical application. Therefore, researchers must implement strategies to address these deficiencies, thereby enhancing the effectiveness of their application in promoting LR.

Oleanolic acid (OA) is a terpenoid mainly found in the leaves of Olea europaea and the fruit of Ligustrum officinale, with anti-inflammatory, antioxidant [108], cardioprotective [109], and hypoglycemic effects [110], and research has shown that OA is a hepatoprotective agent that can alleviate acute liver injury [111], cholestatic liver injury [112], and promote LR. The promotional effects on LR were demonstrated by the ability to promote liver recovery after 70% PH. It is well documented that in the 70% PH mouse model, OA can increase the expression of PCNA in vivo and in vitro [113]. In addition, forkhead box protein O1 (FOXO1), a transcription factor that can regulate cellular processes, and inhibition of its expression promotes cell proliferation [114]. Retinoblastoma-like protein 2 (RBL2) is capable of interacting with other substances to affect cell proliferation, such as the transcription factor E2F, and the inhibition of the RBL2/E2F4 complex can promote cell proliferation [115]. Moreover, cyclin-dependent kinase inhibitor 1B (CDKN1B) is a known cell cycle-dependent kinase inhibitor that inhibits cell proliferation [116], and it has been reported that OA can inhibit the expression of FOXO1, RBL2, and CDKN1B, and promote the proliferation of hepatocytes [113]. Therefore, OA can promote LR following 70% PH through various mechanisms. Ger is a terpenoid natural compound that exists in plants of the Brassicaceae and Geranylgeranyl family, with many important pharmacological activities such as anti-inflammatory [117], antioxidant, and antimicrobial [118]. Studies have demonstrated that Ger has hepatoprotective properties. For example, Ger prevents cyclophosphamide-induced hepatotoxicity [119], improves bisphenol-A-induced liver injury [120], and promotes LR. As we all know, inflammatory cytokines IL-6 and TNF-α are crucial for LR [121], in the 70% PH rat model, Ger can reduce serum ALT levels, increase the expression of IL-6 and TNF-α, as well as the mitotic activity of hepatocytes, and promote LR [122]. In conclusion, the role of Ger in inducing LR after PH has been confirmed, but the specific molecular mechanisms need to be further investigated.

In summary, all these natural products have great potential in promoting LR, and it is interesting to note that different natural products may promote LR through the same mechanism of action, most of them, such as dioscin, PNS, Baicalin, RESV, SolB, UA, and OA, can up-regulate the expression of Cyclins (such as PCNA, CDK2, CDK4, Cyclin D1 and Cyclin E1) to directly promote the proliferation of hepatocytes, and in addition the antioxidant effect of Que, CUR, the regulation of inflammatory factors by DMY, Ger, and the anti-apoptotic effect of DMY, SolB all create favorable conditions for LR.

## Regulatory mechanisms of herbal medicines on LR

**Fig. 2:**
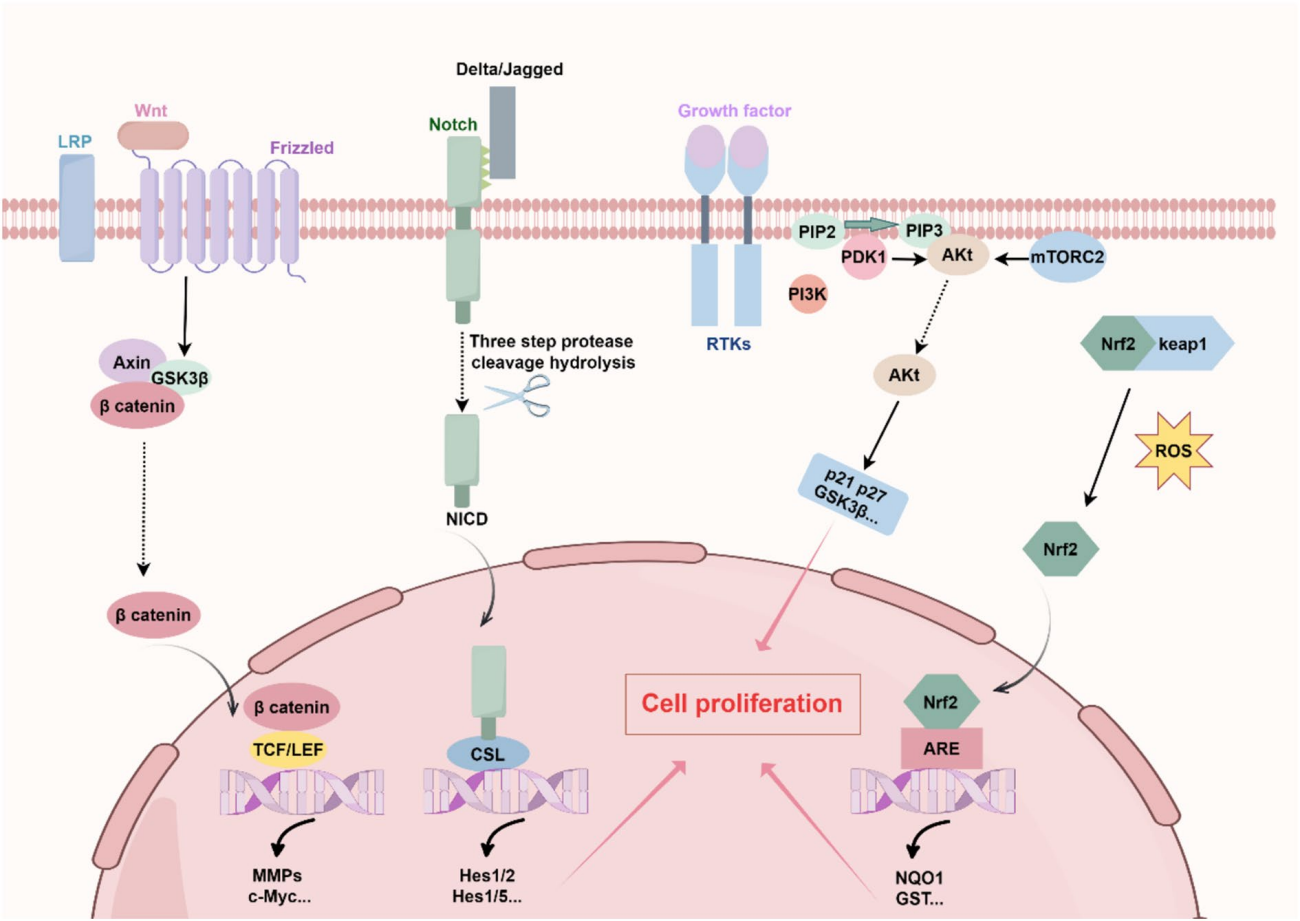
Signaling pathways of natural products for LR. Activation of Wnt/β-catenin, PI3K/Akt, Notch and Nrf2 signalling pathways promotes cell proliferation and is strongly associated with LR. *LR* liver regeneration. (by FigDraw)

In summary, natural product monomers are promotive for LR and can repair damaged liver, and their regulatory mechanisms on LR have received increasing attention. In recent years, several signaling pathways have been identified as being closely associated with LR (Fig. 2).

### Wnt/β-catenin signaling pathway

The Wnt/β-catenin signaling pathway, comprising the Wnt ligand, Frizzled receptor, LRP5/LRP6 co-receptor, Dishevelled (Dvl) protein, glycogen synthase kinase-3β (GSK3β), β-catenin, TCF/LEF transcription factors, Axin protein, and CK1 kinase, regulates cell proliferation, differentiation, and tissue development. This is achieved through the modulation of β-catenin's stability and its transcriptional activity in the nucleus. Notably, activating the Wnt/β-catenin signaling pathway includes the following steps. Firstly, Wnt ligands bind specifically to Frizzled receptors and LRP5/6 co-receptors on the cell membrane, and this binding triggers phosphorylation and dephosphorylation reactions of proteins in the cytoplasm, which can activate β-catenin. Subsequently, β-catenin translocates to the nucleus, where it interacts with TCF/LEF transcription factors and activates the expression of target genes, such as Matrix Metalloproteinases (MMPs) and cellular myelocytomatosis oncogene (c-Myc), which play a role in the regulation of cell proliferation and can thus promote cell proliferation [123]. Therefore, the Wnt/β-catenin signaling pathway is closely associated with LR. Additionally, several natural products have been demonstrated to enhance LR through the modulation of the Wnt/β-catenin signaling pathway. For example, tanshinone IIA (TSA) is a phenanthrenequinone compound extracted from traditional Chinese medicine (TCM) Danshen and β-catenin is a key component of the classic Wnt signaling pathway and has been shown to regulate hepatocyte proliferation, it is well documented that moderate concentrations of TSA can increase the expression of β-catenin, activate the Wnt/β-catenin signaling pathway, and stimulate the proliferation of hepatic oval cells, thus enhancing the repair and regeneration of injured livers and improving LR after orthotopic LT [124]. Furthermore, SNS is a traditional Chinese medicine formula, which can promote liver stem cell differentiation through the Wnt/β-catenin signaling pathway, thereby alleviating liver injury. The study has shown that 14 days after treatment with SNS in PH rats, phosphorylation of GSK3β was significantly increased. GSK3β is a key component of the Wnt signaling pathway, and when the Wnt signaling pathway is inactivated, GSK3β degrades β-catenin, resulting in low intracellular levels of β-catenin. However, when the Wnt signaling pathway is activated, GSK3β is inactivated by phosphorylation, and the level of β-catenin is increased. As a result, the levels of β-catenin and Dvl2 increased, after which β-catenin was transferred to the nucleus and bound to Lymphoid enhancer-binding factor 1 (LEF1) receptor to activate the expression of c-Myc and Cyclin D1, which are related to cell proliferation and differentiation, and activated the Wnt/β-catenin signaling pathway, promoting LR in rats after hepatectomy [125]. Although SNS has shown potential efficacy in promoting LR, its side effects and contraindications must be carefully considered, and its use should adhere strictly to medical guidance. In conclusion, the Wnt/β-catenin signaling pathway represents a highly promising target for enhancing LR, warranting further investigation to fully explore its therapeutic potential.

### PI3K/Akt signaling pathway

The PI3K/Akt signaling pathway is a complex network consisting of the following components: cell surface receptors (e.g., tyrosine kinase receptor), phosphatidylinositol 3 kinase (PI3K) second messenger phosphatidylinositol 3,4,5-trisphosphate (PIP3), protein kinase B (Akt), phosphatidylinositol-dependent protein kinase 1 (PDK1), mechanistic targets of rapamycin complexes 1, 2 (mTORC1, mTORC2), and Phosphatase and Tensin Homolog Deleted on Chromosome 10 (PTEN). Notably, the activation of the PI3K/Akt signaling pathway can affect cell proliferation and involves the following steps. Firstly, growth factors or cytokines bind to cell membrane surface receptors, initiating the activation of PI3K, which phosphorylates phosphatidylinositol 4,5-bisphosphate (PIP2) to form PIP3. PIP3 plays a critical role in recruiting Akt and PDK1 to the plasma membrane. Subsequently, Akt is fully activated through phosphorylation by PDK1 and mTORC2. The activated Akt then regulates various cellular processes, including survival, proliferation, metabolism, and apoptosis, by phosphorylating key substrate proteins such as Bcl2-Associated X (BAX), GSK3β, and cell cycle inhibitors, p21 and p27 [126]. Activation of the PI3K/Akt signaling pathway plays a key role in the early regenerative response after PH. Early studies have shown that inhibition of the PI3K/Akt signaling pathway inhibits hepatocyte replication on the one hand, and leads to structural abnormalities of vacuolization, lipid deposition, and glycogen accumulation of regenerating hepatocytes on the other hand, which attenuates LR [127]. Additionally, it has been reported in studies that some natural products can promote LR through PI3K/Akt signaling pathway. For example, salidroside, a natural product found in the roots of rhodiola rosea, is well documented that salidroside can act on the PI3K/AKT/Gsk3β signaling pathway, significantly reduce ROS production and lipid accumulation, inhibit apoptosis of hepatocytes and promote the proliferation of hepatocytes, which can promote LR [128]. PNS is a kind of saponin natural product extracted from the traditional Chinese herb Panax notoginseng. In the mice model of PH, PNS significantly increased the phosphorylation level of PI3K and Akt, and the activation of Akt initiated the phosphorylation of the downstream target mTOR on the one hand, playing a role in promoting hepatocyte proliferation; on the other hand, Akt initiated the phosphorylation of another target Bcl-2-associated agonist of cell death (Bad), one of the pro-apoptotic proteins of the Bcl-2 family, and in the phosphorylated state, Bad promoted cell survival, in the dephosphorylated state, it exerts the opposite effect to promote apoptosis [129]. Therefore, PNS promotes LR by up-regulating the PI3K/Akt/mTOR and Akt/Bad signaling pathways to promote hepatocyte proliferation and transplantation of hepatocytes for apoptosis [43]. All in all, the therapeutic effects of herbal ingredients through the PI3K/Akt signaling pathway provide more strategies and ideas to promote LR.

### Notch signaling pathway

The Notch signaling pathway plays a crucial role in regulating cell proliferation and apoptosis. It involves several key components, including the Notch receptors (Notch1, 2, 3, and 4), ligands (Jagged-1, Jagged-2, and Delta-1, Delta-3, Delta-4), the DNA-binding protein CSL, and a range of downstream effectors. Normally, activation of the classical Notch signaling pathway involves the following process. Firstly, the Notch protein is transported to the endoplasmic reticulum as a single-chain precursor, where it undergoes glycosylation and is transported to the Golgi for cleavage at the S1 site to form the mature Notch receptor which is transported to the surface of the cell membrane, where it binds to ligands (Jagged/Delta) on the neighboring cell membrane, and this binding triggers the cleavage of the Notch receptor at the S2 site, and the intermediate formed is cleaved by γ-secretase to undergo S3 site cleavage, releasing theNotch1 intracellular domain (NICD). Then, NICD binds to the transcription factor CSL to recruit coactivators that activate the transcription of downstream target genes [130]. Activating the Notch/Jagged signaling pathway is important for LR. On the one hand, activation of the Notch signaling pathway can initiate the transcription of downstream target genes of the Hes and Hey families, which are associated with cell proliferation, and promote hepatocyte proliferation. On the other hand, its activation contributes to the formation of bile ducts, which is an important link in the LR [131]. Furthermore, the researchers demonstrated that in the 2/3 PH rat model, the Notch signaling pathway regulates the cell cycle of proliferating hepatocytes involved in LR and is important for LR processes. After hepatectomy in rats, the Notch signaling pathway was significantly activated, in contrast to the addition of inhibitors of γ-secretase (Notch receptor S3 cleavage essential enzyme for conversion to NICD, a key enzyme in Notch signaling pathway activation) revealed that abnormalities in the S and M phases of the hepatocyte proliferation cycle resulted in delayed LR after PH in rats [132], which indicates that the normal function of the Notch signaling pathway is crucial for the proper progression of LR. Notably, studies have found that natural products can promote LR by activating the Notch signaling pathway. For instance, dioscin, a saponin-like natural product, can significantly increase Notch1 and Jagged1 levels in the 70% PH mice model, upregulate the expression of PS1, and increase PS1-dependent γ-secretase activity, leads to the nuclear translocation of NICD1, and activate the Notch1/Jagged1 signaling pathway, leading to the promotion of the expression of downstream targets associated with cell proliferation: (Hey1, Hes1, EGFR, VEGF) and cell cycle regulatory proteins (CyclinD1, CyclinE1, CDK4, and CDK2) to promote hepatocyte proliferation and LR [32]. Notably, however, whether other natural products can promote LR through the Notch signaling pathway needs to be further explored by researchers.

### Nrf2 signaling pathway

The nuclear factor erythroid 2-related factor 2 (Nrf2) signaling pathway consists of the Nrf2, the kelch-like ECH-associated protein 1 (keap1), the antioxidant response element (ARE), and a series of target genes. The activation of the Nrf2 signaling pathway involves several key processes. Under normal conditions, the Nrf2 protein is tightly bound to Keap1. However, upon oxidative stress or other stimuli, this binding is disrupted. Subsequently, Nrf2 translocates to the nucleus, where it binds to the ARE and activates the transcription and translation of target genes. It has been shown that activation of Nrf2 can advance the mitotic process of cell proliferation through the Cyclin A2 and Wee1/Cdc2/Cyclin B1 pathways, whereas inhibition of Nrf2 delays mitosis in hepatocytes and affects the proliferative process [133].

The Nrf2 signaling pathway has demonstrated potential in promoting LR. Furthermore, natural products have been shown to enhance LR through modulation of Nrf2 activity. For example, CUR and Que, both natural activators of Nrf2, exhibit beneficial effects on LR by promoting cell proliferation, reducing apoptosis, and exerting antioxidant effects [134]. In conclusion, future studies will further elucidate the mechanism of the Nrf2 signaling pathway and provide new insights into LR.

## Self-assembled nanodrug delivery systems

The self-assembled nanodrug delivery system (SANDDS) is an organized nanostructure characterized by a well-defined structure and stability, which is formed through the spontaneous aggregation of molecules via non-covalent interactions, without the need for external intervention. Notably, SANDDS offers several advantages, including simple synthesis, reduced toxicity, and enhanced bioavailability [135].

SANDDS has shown great potential in the biomedical field to enable drugs to perform their optimal roles. For example, compounds such as alkaloids, flavonoids, organic acids, and terpenoids possess multiple functional groups and action sites within their structures, which allow them to self-assemble into self-assembled nanodrug delivery systems (SANDDS). This self-assembly process enhances the bioavailability and therapeutic efficacy of these natural products [136]. For example, CUR by green reprecipitation method synthesized pure CUR nanoparticles (CNPs) without carrier and loaded with cardiolipin LP (liposomes) can improve the poor solubility of curcumin and have more prominent efficacy in Alzheimer's disease (AD) [137].

More importantly, SANDDS can improve the shortcomings of solubility and bioavailability of some of the above-mentioned natural products that promote LR (Table 2), such as DMY, Que, CUR, RESV, RA, and UA, which is very important for the future treatment of liver diseases. DMY, its low solubility, poor permeability, unstable nature, and rapid metabolism in vivo lead to poor bioavailability and unsatisfactory pharmacodynamics [138] [139]. DMY-MS self-assembled polymeric micelles formed by self-assembly of DMY with Polyethylene glycol-15-hydroxystearate (Solutol®HS15) as a carrier significantly improved the solubility, oral bioavailability, and anti-alcohol resistance of DMY and had a slow-release [140]. CUR also has the disadvantages of poor solubility and stability, it can be self-assembled and encapsulated in H. pluvialis protein (HPP)-galactose (GAL) nano complex (HPP-GAL) nanoparticles by hydrogen bonding to synthesize HPP-GAL-CUR nano preparations, which improves the stability and bioavailability of CUR, and in the ALD mice model, HPP-GAL-CUR exhibits good liver-targeting properties due to the ability of GAL itself to accumulate in the liver, and is more effective than free CUR for the treatment of ALD in mice [141]. SANDDS can also solve the challenges of RESV applications, RESV has poor water solubility and low chemical stability and is susceptible to chemical degradation stimulated by exogenous environmental stresses [142, 143]. Notably, the solubility and stability of RESV were improved by encapsulating RESV in nanoparticulate Res NPs formed by self-assembly of α-lipoic acid (α-LA), lactobionic acid (LA), and glycogen (Gly) into nanocarriers. In addition, in the NAFLD mice model, Res NPs were able to target the liver with better therapeutic efficacy compared to the free form of RESV [144] and facilitate the future use of RESV in the treatment of clinical diseases, including LR. Furthermore, RA as a key polyphenolic antioxidant can promote LR, but its poor stability as well as low bioavailability have greatly limited its translation into clinical applications. Nanoparticles of PRA NPs synthesized by self-assembled RA, 1,4-phenylene diboronic acid (PBA), and glycerol monooleate (GMO), improved the stability of RA and were able to be targeted to the liver with increased antioxidant activity relative to free RA in the mouse model of acute liver injury [145]. UA is also one of the natural products mentioned above that promotes LR, researchers developed an amphiphilic self-assembled nanomedicine consisting of UA, LA, and low-polyamidoamine (low-PAMAM) dendrimer: UA-G0/G1-LA, which solved the problem of low solubility of UA, and in the H22 mice model, the positive electric charge and nano size of UA-G0/G1-LA helped to be targeted to the tumor site and effectively inhibited the growth of liver tumors, which significantly improved the retention time and bioavailability of UA in vivo [146]. In addition, TSA is a natural product that can promote LR [124], but some limitations exist in its clinical use, such as poor solubility, poor photothermal stability, short elimination half-life, poor hepatic targeting, and strong hepatic first-pass effect [147], which greatly reduces its bioavailability. mPEG-DCA is a drug carrier that can control the release of the drug, prolong the circulating time, and improve the drug accumulation (EPR effect) in the tumor. Therefore, mPEG-DCA was used as a carrier for co-loading TSA and GA to synthesize nano preparations, which improved the solubility and chemical stability of TSA and GA. More importantly, it showed good tumor targeting and biocompatibility in H22 tumor-bearing mice, prolonged the mean residence time (MRT) of TSA in mice, and had a better therapeutic efficacy [148].

**Table 2:** Application of different types of SANDDS to natural products that promote LR

Natural products	Types of SANDDS	Delivery site/Disease	Characteristics of SANDDS	References
Dihydromyricetin (DMY)	Self-assembled DMY-loaded Solutol®HS15 micelles	Stomach and liver tissue / Alcohol-induced liver and stomach injury	Solubility and bioavailability of DMY**↑** Possessed good stability, biocompatibility, and slow-release properties	[140]
Curcumin (CUR)	HPP-GAL-CUR nanoparticles	Liver tissue/ Acute alcoholic liver damage	Solubility and stability of CUR**↑** Possessed liver targeting properties	[141]
Resveratrol (RESV)	Resveratrol glycogen-based nanoparticles	HepG2 cells and liver tissue/ Nonalcoholic fatty liver disease	Bioavailability of RESV**↑** Possessed liver targeting properties	[144]
Rosmarinic acid (RA)	PRA NPs nanoparticles synthesized by self-assembly of RA,1,4-phenyldiboronic acid (PBA) and glyceryl monooleate (GMO)	LO2 cells and liver tissue/Acute liver injury	Antioxidant effects and stability of RA**↑** Possessed liver targeting properties	[145]
Ursolic acid (UA)	Lactobionic acid-modified amphiphilic low-generation PAMAM dendrimer-UA coupling for self-assembled nanoparticles: UA-G0/G1-LA	SMMC7721 hepatocellular carcinoma cells / Hepatocellular carcinoma	Solubility and bioavailability of UA**↑** Retention time in vivo**↑** Possessed liver targeting properties	[146]
Tanshinone IIA (TSA)	TSA and GA co-loaded self-assembled nanoparticles	HepG2 cells and liver tissue/ Hepatoma	Solubility, stability and biocompatibility of TSA↑ Possessed liver targeting and Slow-release properties	[148]

Taken together, the application of SANDDS has the potential to address the limitations of natural products and enhance their therapeutic efficacy, offering promising prospects for the medical field.

## Future prospects

**Fig. 3:**
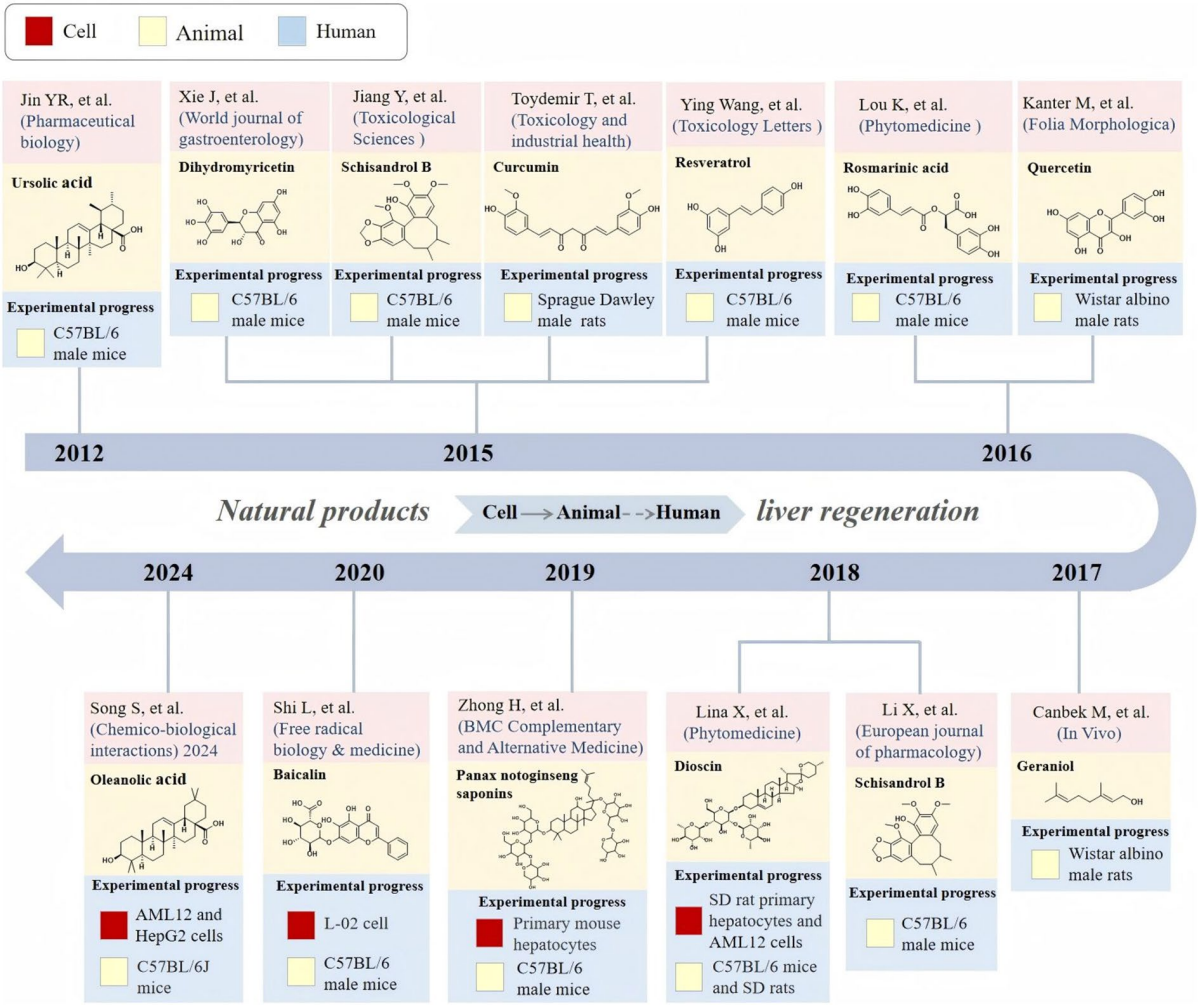
Experimental progress of natural products on LR. This timeline illustrates the experimental progress of multiple natural products in LR research, covering cell, animal, and human (no human experiments are currently in progress) studies from 2012 to 2024. LR: liver regeneration

Altogether, natural products including ingredients of TCM are rich resources for the discovery of new medicines, and natural products exhibit obvious anti-inflammatory, antioxidant, and hepatoprotective effects, garnering substantial research interest. This review summarizes the research progress on natural products, including TCM, in promoting LR. Numerous studies have demonstrated that natural products can enhance LR by modulating various signaling pathways. Although studies have confirmed the ability of natural products to promote LR, this ability has only been verified in animal experiments, and no substantial progress has been made in relevant clinical trials (Fig. 3), natural products have the advantages of low toxicity, wide sources, and diverse structures, and some marketed natural product formulations can promote hepatocyte repair and regeneration, such as the Five Spirit pill (TCM) and Huganning Tablets, they can also be transformed into lead compounds [149] for the development of new drugs to promote LR based on their diverse structural properties, thus these natural products have potential for clinical applications. Moreover, the exploration of the targets of action of natural products on LR is still limited, and the dynamic changes of gene expression, protein changes, and metabolic pathways in the process of LR promotion by natural products can be comprehensively analyzed by multi-omics technology [150, 151] to uncover new targets and molecular mechanisms. Therefore, it remains a challenge in the future to translate the LR-promoting effects of these natural products from animal studies to human applications and to exploit their novel targets for LR.

However, challenges related to improving their bioavailability and minimizing potential side effects remain unresolved and need to be addressed in both current and future research. One approach to enhancing the bioavailability of natural products while minimizing side effects is the use of targeted drug delivery systems (DDS) [152, 153]. These systems allow for precise delivery of drugs to the intended site of action. Among DDS, nanoparticle-based drug carriers are commonly employed. By encapsulating active ingredients within these nano carriers, their solubility and bioavailability are improved, which in turn enhances their therapeutic efficacy [154]. SANDDS have garnered increasing attention from scientists due to their advantageous properties, these spontaneous nano-formulations, formed through non-covalent interactions, are relatively simple to prepare and exhibit superior biodegradability and biocompatibility compared to traditional drug nano-formulations [136]. As a result, they hold the potential for enhancing the efficacy of drug treatments for various diseases, including liver disease. For example, Ger is an effective natural product against hepatocellular carcinoma (HCC), but its poor solubility has greatly hindered its clinical application. The multiple bioresponsive self-assembled nano drug delivery system (HSSG) developed by using self-assembly technology to couple Ger with hyaluronic acid (HA) improves the therapeutic efficacy of Ger against HCC, realizes the targeted and controlled release of the drug, and reduces its toxicity [155]. In conclusion, SANDDS is anticipated to enhance the bioavailability and hepatic targeting of natural products that promote LR, with the goal of minimizing adverse effects and improving therapeutic efficacy.

In addition, the use of artificial intelligence (AI) technology to directly screen for drugs that can promote LR from a broad range of natural product molecules holds significant potential for advancing LR research. As AI technology becomes increasingly widespread [156], it is important to note that AI can analyze large volumes of biomedical data to identify new therapeutic targets, including those related to cancer [157], and this approach not only accelerates the target identification process but also improves the accuracy and effectiveness of drug design. PandaOmics is a platform that integrates artificial intelligence and bioinformatics technologies to identify therapeutic targets and biomarkers for various diseases. It utilizes a range of disease-specific models to rank potential targets, aiding in the screening of therapeutic targets. This platform has previously been used to identify targets for the treatment of amyotrophic lateral sclerosis [158]. Taken together, AI demonstrates significant potential in screening drug targets. Moving forward, we anticipate utilizing various AI platforms to identify targets and natural products that can promote LR.

In a word, natural products including TCM have a promising future in LR, and corresponding research and development are in progress, but the acceleration of the clinical translation of natural products to LR, the discovery of natural products that can act on new targets of LR and the reduction of toxic side effects of natural products in LR remain an important task.

## Conclusion

In summary, natural products including TCM have shown great potential and unique advantages in promoting LR As research progresses, natural products are expected to offer more effective therapeutic options for enhancing LR. However, improving the targeting capabilities of these products remains a critical challenge. We believe that loading natural products onto SANDDS can be utilized to localize their targeting effects on hepatocytes and promote LR, and that screening for targets and drugs to promote LR using AI and multi-omics technology has unlimited potential in the future. Consequently, it is essential for researchers to engage in sustained and comprehensive studies in this field to drive substantial progress.

## Data Availability

No data was used for the research described in the article.

## Abbreviations

α-LA: α-Lipoic acid
Aa: Aurantiamide acetate
AD: Alzheimer's disease
AFLD: Alcoholic fatty liver disease
AI: Artificial intelligence
Akt: Protein kinase B
ALD: Alcoholic liver disease
ALP: Alkaline phosphatase
ALT: Alanine aminotransferase
APAP: Acetaminophen
ARE: Antioxidant response element
AST: Aspartate aminotransferase
BA: Bile acids
Bad: Bcl-2-associated agonist of cell death
BAX: Bcl2-Associated X
Bcl-2: B-cell lymphoma-2
CDKs: Cell cycle protein-dependent kinases
CDK2: Cyclin-dependent kinase 2
CDK4: Cyclin-dependent kinase 4
CDKN1B: Cyclin-dependent kinase inhibitor 1B
C/EBPβ: CCAAT/enhancer-binding protein β
c-Myc: Cellular myelocytomatosis oncogene
CNPs: Curcumin nanoparticles
CUR: Curcumin
Cyclins: Cell cycle proteins
Cyclin D1: Cell cycle protein D1
Cyclin E1: Cell cycle protein E1
CYP450: Cytochrome P450
DDS: Drug delivery systems
DMY: Dihydromyricetin
Dvl: Dishevelled
EGF: Epidermal growth factor
FOXO1: Forkhead Box protein O1
GAL: Galactose
Ger: Geraniol Gly: Glycogen
GMO: Glycerol monooleate
GSH: Glutathione
GSK3β: Glycogen synthase kinase 3β
HA: Hyaluronic acid
HCC: Hepatocellular carcinoma
HGF: Hepatocyte growth factor
Hif-1α: Hypoxia inducible factor-1-α
HPP: H. pluvialis protein
HSSG: Multiple bioresponsive self-assembled nano drug delivery system
IL-1β: Interleukin-1β
IL-18: Interleukin-18
IL-6: Interleukin-6
I/R: Ischemia–reperfusion
keap1: Kelch-like ECH-associated protein 1
LA: Lactobionic acid
LEF1: Lymphoid enhancer-binding factor 1
Low-PAMAM: Low-Polyamidoamine
LR: Liver regeneration
LT: Liver transplantation
LP: Liposomes
MDA: Malondialdehyde
MMPs: Matrix Metalloproteinases
MRT: Mean residence time
mTORC1: Mechanistic targets of rapamycin complexes 1
mTORC2: Mechanistic targets of rapamycin complexes2
NAFLD: Non-alcoholic fatty liver disease
NAPQI: N-acetyl-p-benzoquinone imine
NASH: Nonalcoholic steatohepatitis
NE: Norepinephrine
NICD: Notch1 intracellular domain
NLRP3: NOD-like receptor pyrin domain containing 3
Nrf2: Nuclear factor erythroid 2-related factor 2
OA: Oleanolic acid
P: Palmitin
PBA: 1,4-Phenylene diboronic acid
PCNA: Proliferating cell nuclear antigen
PDK1: Phosphatidylinositol-dependent protein kinase 1
PH: Partial hepatectomy
PIP2: Phosphatidylinositol 4,5-bisphosphate
PIP3: Phosphatidylinositol 3,4,5-trisphosphate
PI3K: Phosphatidylinositol 3 kinase
PNS: Panax notoginseng saponin
PTEN: Phosphatase and Tensin Homolog Deleted on Chromosome 10
Que: Quercetin
RA: Rosmarinic acid
RBL2: Retinoblastoma-like protein 2
RESV: Resveratrol
ROS: Reactive oxygen species
SA: Scutebarbatine A
SANDDS: Self-assembled nanodrug delivery systems
SNS: SiNiSan
SOD: Superoxide dismutase
SolB: Schisandrin B
TCM: Traditional Chinese medicine
TGF-α: Transforming growth factor-α
TGF-β: Transforming growth factor-β
TNF-α: Tumor necrosis factor-α
TPGS: D-α-Tocopherol polyethylene glycol 1000 succinate
TSA: Tanshinone IIA
UA: Ursolic acid
VEGF: Vascular endothelial growth factor
